# Paraneoplastic Hypoglycaemia in a Patient With Solitary Fibrous Tumor of Pleura

**DOI:** 10.1002/rcr2.70275

**Published:** 2025-07-21

**Authors:** Asmita A. Mehta, Valiyaparambil Pavithran Praveen, Valathara Pradeep Lakshmi Priya, Liya Anil, Vishnu Nair

**Affiliations:** ^1^ Department of Respiratory Medicine Amrita Institute of Medical Sciences and Research Kochi India; ^2^ Department of Endocrinology and Diabetes Amrita Institute of Medical Sciences and Research Kochi India

**Keywords:** Doege‐potter syndrome, hypoglycemia, insulin‐like growth II, solitary fibrous tumours

## Abstract

Solitary fibrous tumours (SFTs) are rare spindle cell neoplasms, predominantly benign, comprising less than 2% of all soft tissue masses. In rare cases, SFTs can lead to Doege‐Potter syndrome, a paraneoplastic condition characterised by hypoglycaemia due to elevated levels of insulin‐like growth factor II (IGF‐II). Patients typically present with symptomatic hypoglycaemia related to this hormonal dysregulation. We report the case of a 70‐year‐old man who presented with weight loss, cough, fatigue, tingling, and episodes of confusion associated with recurrent hypoglycaemia. Chest radiography revealed a large soft tissue mass in the right lower hemithorax, and CT‐guided biopsy confirmed tumour infiltration in the basal segment of the right lower lobe.

## Graphical abstract

A 70‐year‐old man with recurrent hypoglycaemia was diagnosed with Doege‐Potter syndrome due to a giant solitary fibrous tumour of the pleura. Complete surgical resection of the 30 × 21 × 14 cm mass resolved hypoglycaemic episodes and restored functional stability.

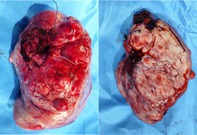

## 1 Introduction

Doege‐Potter syndrome is a rare paraneoplastic syndrome characterised by symptomatic hypoglycaemia associated with a solitary fibrous tumour (SFT). These tumours secrete prohormone forms of insulin‐like growth factor II (IGF‐II), which suppress growth hormone and reduce the synthesis of IGF‐binding proteins. This results in increased binding of IGF‐II to insulin receptors, leading to enhanced peripheral glucose uptake and subsequent hypoglycaemia [1]. We present a case of a patient with an SFT whose daily functioning was significantly affected by recurrent hypoglycaemia.

## 2 Case Report

A middle‐aged man presented with complaints of cough, fatigue, tingling in the fingers, mild hemoptysis, and unintentional weight loss of 5 kg over 2 months, along with a loss of appetite. He also reported episodes of mild shivering and numbness in the extremities, particularly during the early morning hours. He had been previously diagnosed with recurrent hypoglycaemic episodes of unknown origin and was referred to our centre for further evaluation.

On admission, laboratory investigations revealed the following: haemoglobin (Hb) 10.8 g/dL (reference: 13.5–17.5 g/dL), white blood cell (WBC) count 4.8 × 10^9^ cells/L (4.0–11.0 × 10^9^ cells/L), red blood cell (RBC) count 3.64 × 10^12^ cells/L (4.5–5.9 × 10^12^ cells/L), platelet count (PLT) 170 × 10^9^ cells/L (150–400 × 10^9^ cells/L), neutrophils 50.6% (40%–70%), lymphocytes 33.1% (20%–40%), C‐reactive protein (CRP) 0.57 mg/L (< 5 mg/L), serum creatinine 62.8 μmol/L (62–106 μmol/L), and negative serum ketones. Thyroid function tests were within normal limits: T4 was 17.77 pmol/L (10–22 pmol/L), thyroid‐stimulating hormone (TSH) was 3.86 mIU/L (0.4–4.0 mIU/L), and HbA1c was 31.77 mmol/mol (< 42 mmol/mol).

**FIGURE 1:**
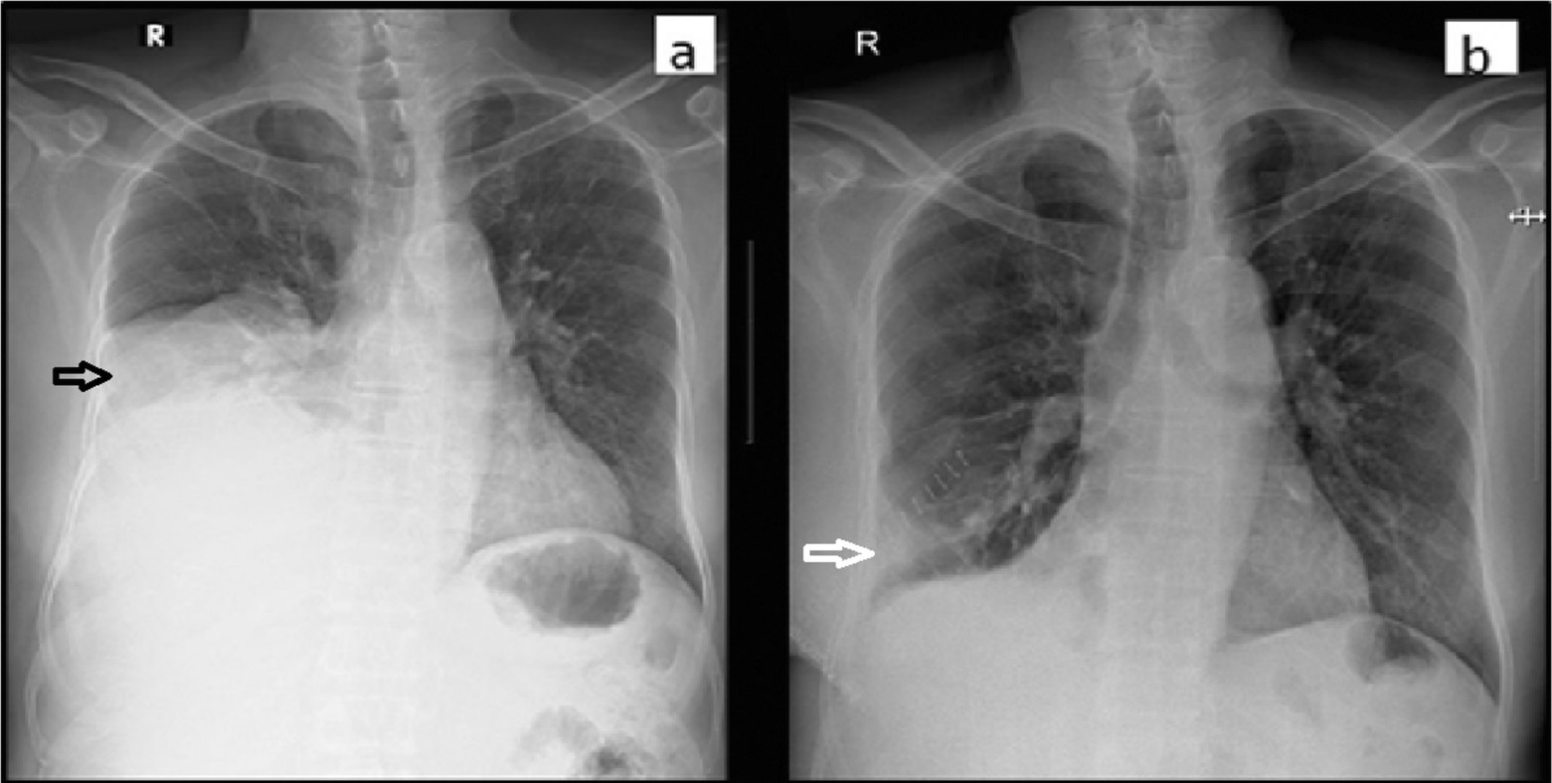
Showing the comparison of chest radiography (a) chest X‐ray before the surgery showing right side middle and lower homogenous opacity in right hemithorax. (b) chest X‐ray after tumour debulking.

**FIGURE 2:**
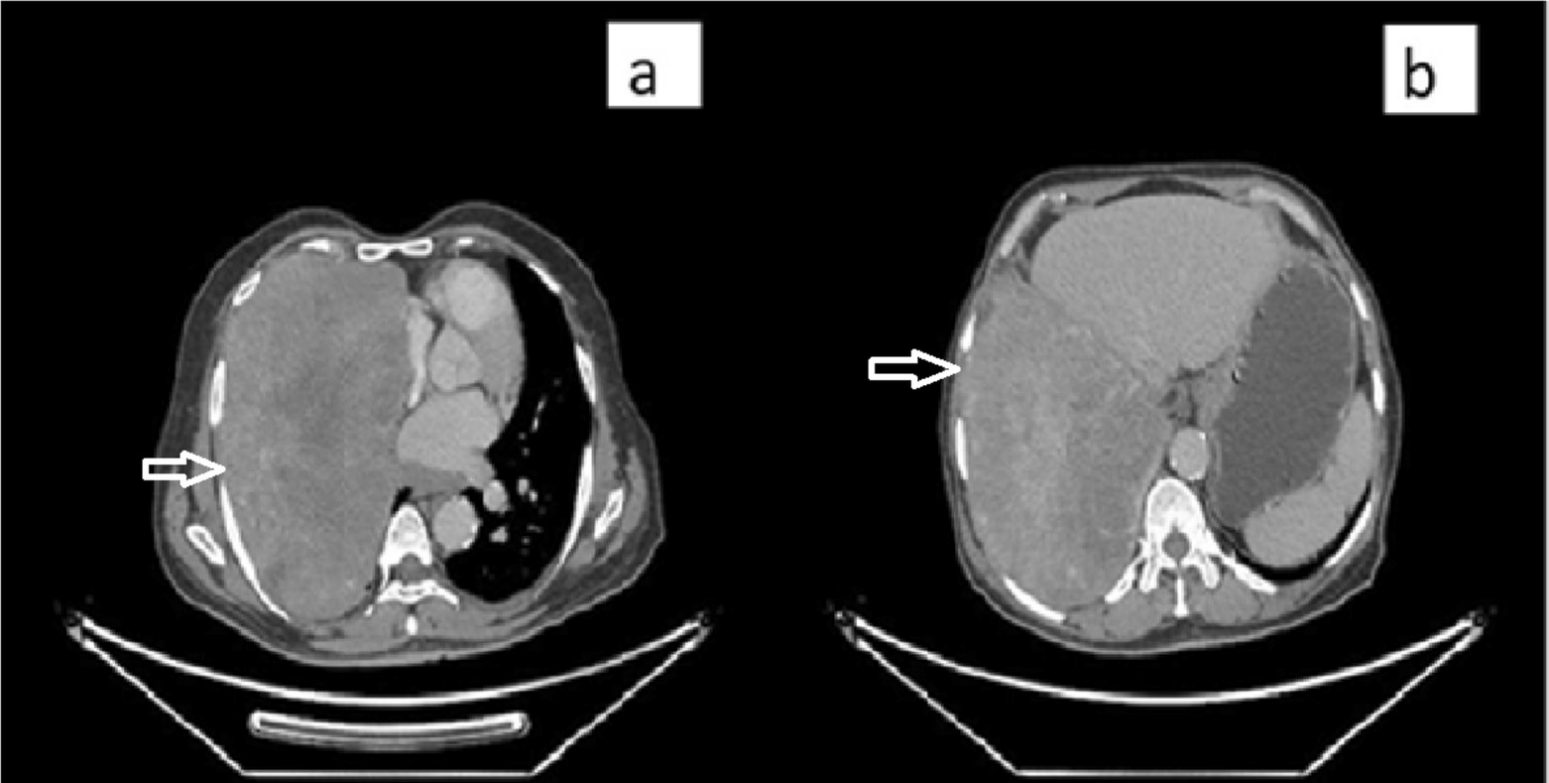
Computed tomography showing large well defined heterogeneously enhancing soft tissue mass lesions 20 × 13 x 19 cm with central scaring in the right hemithorax, causing complete collapse consolidation of the right middle lobe and partial collapse consolidation of the right middle lobe (a) huge heterogeneous mass pointing towards the heart not invading and are well capsulated (b) huge heterogeneous mass pointing towards the liver not invading and are well capsulated.

Chest radiography revealed haziness in the right middle and lower lobes (Figure 1). A contrast‐enhanced CT scan of the chest showed a large, well‐defined soft tissue density mass measuring 204 × 124 × 162 mm in the right lower hemithorax, causing collapse consolidation of the right middle and lower lobes (Figure 2). The mass exhibited foci of calcification and heterogeneous enhancement. Its arterial supply originated from the abdominal aorta. Medially, the lesion exerted compressive effects on the superior vena cava, right atrium, and inferior vena cava.

During hospitalisation, the patient experienced episodes of hypoglycaemia with glucose levels as low as 40 mg/dL. An endocrinology consultation was sought for further management of hypoglycaemia.

On admission, laboratory investigations were performed to evaluate the aetiology of the patient's recurrent hypoglycaemia. The Table 1 summarises the relevant pre‐operative biochemical parameters.

**TABLE 1:** Laboratory investigations for hypoglycaemia.

Laboratory parameters	Pre‐operative value	Reference range
Glucose	2.25 mmol/L	4.44–11.11 mmol/L
Serum cortisol	8.82 mcg/dL	5–25 mcg/dL (8: 00 AM)
Serum C peptide (glucose 40 mg%)	0.040 mcg/L	0.364–1.456 mcg/L
Insulin growth factor 1	5.51 mcg/L	14.95–39.91 mcg/L
Growth hormone	0.05915 mcg/L	0.0065–0.39 mcg/L
Serum insulin (glucose 40 mg%)	< 1.2 pmol/L	12.0–149.4 pmol/L

An inpatient laboratory assessment ruled out insulinoma, as insulin and C‐peptide levels were not elevated in the context of spontaneous hypoglycemia (Table 1). This raised a strong suspicion of hypoglycemia due to non–insulin‐mediated mechanisms. Since IGF‐II assays are not available in our region, we relied on indirect methods to establish the aetiology. When the patient's glucose level dropped below 45 mg/dL, 1 mg of intravenous glucagon was administered. Subsequent blood glucose measurements at 10, 20, and 30 min were 3.48, 4.98, and 5.66 mmol/L, respectively (normal range: 3.9–5.5 mmol/L). The observed rise in glucose following glucagon suggested a pre‐receptor or post‐receptor pathology involving insulin or insulin‐like activity as the cause of hypoglycemia.

**FIGURE 3:**
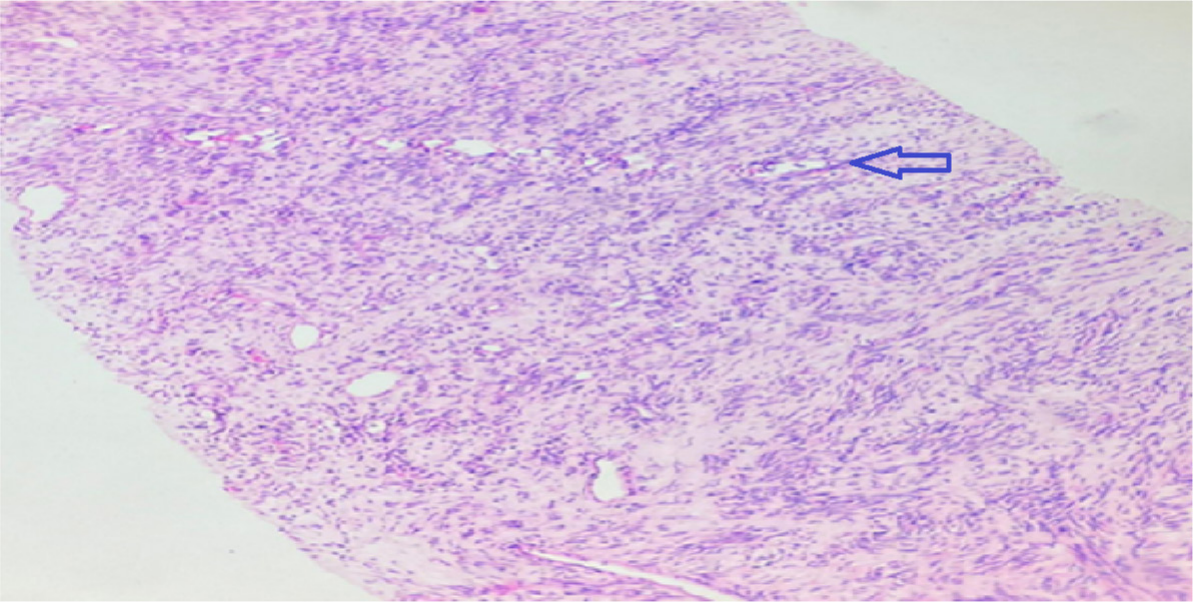
Haematoxylin and eosin staining showing a high‐grade spindle cell sarcoma.

Given the low levels of insulin, IGF‐I, and C‐peptide, combined with a positive glucagon stimulation test, the possibility of an IGF‐II–secreting tumour was considered. A CT‐guided lung biopsy was performed to confirm the diagnosis. Histopathological examination revealed a neoplasm composed of spindle cells arranged in fascicular patterns with elongated vesicular nuclei, moderate eosinophilic cytoplasm, and inconspicuous nucleoli (Figure 3).

Immunohistochemistry of the biopsy showed strong nuclear positivity for STAT6, focal positivity for CD34 in spindle cells, and a Ki‐67 proliferation index of 5%–6% (Figure 3). Methylprednisolone 5 mg once daily was initiated as prophylaxis for hypoglycaemia. Following clinical improvement, the dose was reduced to 2.5 mg daily. A final diagnosis of a solitary fibrous tumour with low‐grade spindle cell morphology was established.

**FIGURE 4:**
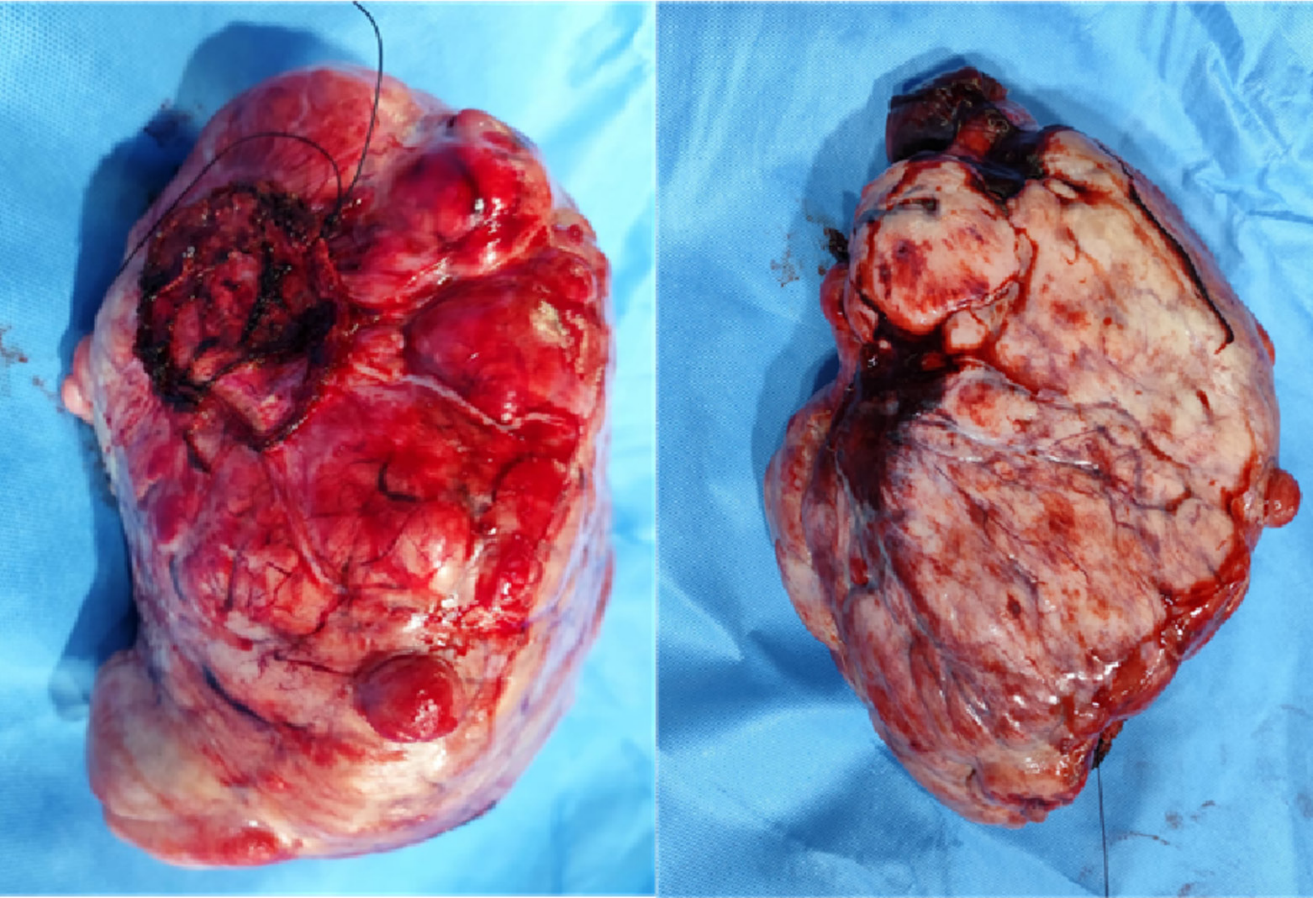
Tumour resection. Gross specimen shows 30 × 21 × 14 cm well encapsulated mass.

The patient underwent mediastinal tumour excision via right posterolateral thoracotomy, along with prolene mesh repair of the right diaphragm. Intraoperatively, a firm, encapsulated mass measuring 30 × 20 × 14 cm was identified, occupying the right pleural cavity and arising from the dome of the right diaphragm, with infiltration into the middle and lower lobes of the right lung (Figure 4). Due to the tumour's large size, debulking was performed.

Following tumour resection, the patient's hypoglycaemic episodes resolved, and corticosteroids were gradually tapered under endocrinology supervision. Given the tumour's size and location, and the risk of recurrence, he was advised to undergo image‐guided radiation therapy. A multidisciplinary tumour board review supported the decision to proceed with adjuvant radiation. The patient completed 25 fractions of radiation therapy and is currently stable.

The patient remained hemodynamically stable and was discharged home. Timely and accurate diagnosis, followed by optimised therapy, led to complete recovery. He has been advised to closely monitor his blood glucose levels at home.

## 3 Discussion

Solitary fibrous tumours (SFTs) are rare mesenchymal neoplasms, most commonly arising in the pleura but also reported in extrapleural sites. While typically benign, 10%–20% may exhibit malignant behaviour. The incidence of SFTs is estimated at 0.35–2.8 per 100,000 individuals. Among these, approximately 30% originate in the pleura, 30% in the abdomen, 20% in the head and neck, and 20% in bones or soft tissues [2, 3, 4]. The pathognomonic NAB2–STAT6 gene fusion plays a central role in tumourigenesis, and STAT6 immunopositivity remains a reliable diagnostic marker [5].

Doege‐Potter syndrome is a paraneoplastic condition seen in 5%–10% of SFTs, particularly those arising from the pleura [4, 5]. It is characterised by refractory, fasting hypoglycaemia due to tumour secretion of insulin‐like growth factor II (IGF‐II) [6]. The condition is often underdiagnosed unless there is a high index of suspicion in patients with large thoracic masses and unexplained hypoglycaemia.

In DPS, tumours secrete high molecular weight “big” IGF‐II, which bypasses normal IGF‐binding protein regulation. Normally, IGF‐II forms a ternary complex with IGF‐binding protein 3 (IGF‐BP3) and the acid‐labile subunit, limiting its bioavailability. In contrast, big IGF‐II forms binary complexes that can freely cross the endothelium, bind insulin and IGF receptors, and enhance peripheral glucose uptake. This leads to hypoglycaemia, suppressed glucagon secretion, reduced hepatic glucose output, and decreased levels of growth hormone and IGF‐I due to feedback inhibition [2, 5].

Our patient had hypoglycaemia (2.25 mmol/L), suppressed insulin (< 1.2 pmol/L), low C‐peptide (0.040 mcg/L), and reduced IGF‐I (5.51 mcg/L), with a positive glucagon stimulation test—all consistent with NICTH. After complete surgical excision of the tumour, the hypoglycaemic episodes resolved, and steroid therapy was successfully tapered off, indicating biochemical cure. Post‐operatively, serial blood glucose levels normalised (random blood glucose of 6.6 mmol/L). The patient remained euglycaemic off corticosteroids, and no further hypoglycaemic episodes were reported.

Surgical excision remains the definitive treatment for DPS. In our case, the tumour measured 30 × 20 × 14 cm, caused compression of mediastinal structures, and resulted in daily hypoglycaemic episodes. These features necessitated surgical intervention. Radiation therapy was used postoperatively due to concerns of residual disease and risk of recurrence. The management strategy aligns with current recommendations and is supported by similar outcomes reported in the literature, including a recent case series by Suzuki et al. [7] which described recurrence after long asymptomatic periods in incompletely resected pleural SFTs.

We acknowledge the limitation of not being able to directly measure IGF‐II levels due to unavailability of the assay in our setting. However, indirect parameters (low IGF‐I, low insulin/C‐peptide, positive glucagon test) and clinical resolution post‐tumour excision strongly support the diagnosis.

This case underscores the importance of considering Doege‐Potter syndrome in patients presenting with large intra‐thoracic masses and unexplained hypoglycaemia. Timely diagnosis and surgical resection are key to resolution. Clinicians should remain vigilant for this rare but life‐threatening condition, especially in regions with limited diagnostic resources. Awareness of DPS can prevent delays in treatment and reduce the risk of morbidity associated with severe hypoglycaemia.

## Author Contributions

Conceptualization: A.A.M. Methodology: A.A.M. Software: A.A.M. Formal analysis: A.A.M. and V.P.L.P. Investigation: V.P.P., A.A.M., L.A., and V.N. Data curation: A.A.M., L.A., and V.N. Writing – original draft preparation: A.A.M. and V.P.L.P. Writing – review and editing: V.P.P., A.A.M., and V.P.L.P. All authors have read and agreed to the published version of the manuscript.

## Consent

The authors declare that written informed consent was obtained for the publication of this manuscript and accompanying images using the consent form provided by the Journal.

## Conflicts of Interest

The authors declare no conflicts of interest.

## Data Availability

The data that support the findings of this study are available from the corresponding author upon reasonable request.
