# Correction to “Paraneoplastic Hypoglycaemia in a Patient With Solitary Fibrous Tumor of Pleura”

**DOI:** 10.1002/rcr2.70317

**Published:** 2025-08-15

A. A. Mehta, V. P. Praveen, V. P. Lakshmi Priya, L. Anil, and V. Nair, “Paraneoplastic Hypoglycaemia in a Patient With Solitary Fibrous Tumor of Pleura,” *Respirology Case Reports* 13 no. 7 (2025): e70275, https://doi.org/10.1002/rcr2.70275.

Below Figure 3 caption is incorrect:

Haematoxylin and eosin staining showing a high‐grade spindle cell sarcoma.

It should be corrected to:

Histopathology (H&E stain, 40x) showing spindle cells arranged in fascicles with elongated vesicular nuclei and eosinophilic cytoplasm, consistent with solitary fibrous tumour.

We apologize for this error.

